# Resting Heart Rate Is a Risk Factor for Mortality in Chronic Obstructive Pulmonary Disease, but Not for Exacerbations or Pneumonia

**DOI:** 10.1371/journal.pone.0105152

**Published:** 2014-08-26

**Authors:** Miriam J. Warnier, Frans H. Rutten, Anthonius de Boer, Arno W. Hoes, Marie L. De Bruin

**Affiliations:** 1 Julius Center for Health Sciences and Primary Care, University Medical Center Utrecht, Utrecht, The Netherlands; 2 Division of Pharmacoepidemiology and Clinical Pharmacology, Utrecht Institute for Pharmaceutical Sciences, Utrecht University, Utrecht, The Netherlands; University of Florida, United States of America

## Abstract

**Background:**

Although it is known that patients with chronic obstructive pulmonary disease (COPD) generally do have an increased heart rate, the effects on both mortality and non-fatal pulmonary complications are unclear. We assessed whether heart rate is associated with all-cause mortality, and non-fatal pulmonary endpoints.

**Methods:**

A prospective cohort study of 405 elderly patients with COPD was performed. All patients underwent extensive investigations, including electrocardiography. Follow-up data on mortality were obtained by linking the cohort to the Dutch National Cause of Death Register and information on complications (exacerbation of COPD or pneumonia) by scrutinizing patient files of general practitioners. Multivariable cox regression analysis was performed.

**Results:**

During the follow-up 132 (33%) patients died. The overall mortality rate was 50/1000 py (42–59). The major causes of death were cardiovascular and respiratory. The relative risk of all-cause mortality increased with 21% for every 10 beats/minute increase in heart rate (adjusted HR: 1.21 [1.07–1.36], p = 0.002). The incidence of major non-fatal pulmonary events was 145/1000 py (120–168). The risk of a non-fatal pulmonary complication increased non-significantly with 7% for every 10 beats/minute increase in resting heart rate (adjusted HR: 1.07 [0.96–1.18], p = 0.208).

**Conclusions:**

Increased resting heart rate is a strong and independent risk factor for all-cause mortality in elderly patients with COPD. An increased resting heart rate did not result in an increased risk of exacerbations or pneumonia. This may indicate that the increased mortality risk of COPD is related to non-pulmonary causes. Future randomized controlled trials are needed to investigate whether heart-rate lowering agents are worthwhile for COPD patients.

## Introduction

Chronic obstructive pulmonary disease (COPD) is a leading cause of morbidity and mortality worldwide [1]. The World Health Organization estimates that by 2020, COPD will be the third most common cause of death in the world [1], [2]. Even when COPD is optimally treated, patients periodically experience exacerbations, resulting in decrease of lung function and quality of life, and often requiring costly hospitalization [3]. COPD and cardiovascular disease share important pathophysiological pathways, and cigarette smoking is a prominent risk factor for both clinical important pulmonary and cardiovascular events. Importantly, in patients with COPD, hospitalisations and deaths are more often caused by cardiovascular events than by respiratory failure [4].

Previous studies showed that patients with COPD had a significantly higher resting heart rate than patients without COPD [5]–[7]. The etiology of the increased heart rate in COPD patients is not yet completely understood, but it may be related to deconditioning, medication use, e.g. β_2_-agonists [8], [9], and undiscovered cardiovascular disease [10], [11]. In observational studies, beta-blockers seemed to have a beneficial effect on all-cause mortality and the risk of exacerbations in patients with a diagnosis of COPD [11]. One of the mechanisms underlying this effect could be the heart rate reducing capacities of beta-blockers.

Whether the increased heart rate in COPD patients is associated with mortality and/or exacerbations or pneumonia is unclear. Therefore, we determined whether resting heart rate was associated with cardiovascular, respiratory, and all-cause mortality, but also with non-fatal pulmonary complications (e.g. pneumonia or exacerbation of COPD) in patients with chronic obstructive pulmonary disease.

## Materials and Methods

### Settings and study design

A prospective cohort study was performed in 405 patients recruited between April 2001 and June 2003 from the vicinity of Utrecht, The Netherlands. The patients, aged 65 years or older, had a general practitioner's diagnosis of COPD (International Classification of Primary Care [IPCP] code R91 [chronic bronchitis] or R95 [COPD or emphysema]). The cohort was described in detail elsewhere [10], [12]. In short, all patients underwent extensive investigations, including electrocardiography (ECG) and pulmonary function testing. Patients with a cardiologist-confirmed diagnosis of heart failure (5.7% of the participants) were excluded because the main aim of the original study was to assess the prevalence of unrecognised heart failure. The Medical Ethics Committee of the University Medical Center Utrecht, the Netherlands, approved the study and all participants gave written informed consent.

### Electrocardiography

A standard resting 12-lead ECG was recorded (GE electronics, San Diego, California). To obtain the mean RR interval length, hard copy ECGs were scanned and converted to digital ECG files (ECGScan Version 3.0, AMPSLLC, New York) [13]. Subsequently the ECGs were processed by a digital calliper software system (CalECG, Version 1.0, AMPSLLC, New York) [14]. To determine the heart rate, the following formula was used: heart rate = 60/RR.

### Follow-up

In order to obtain information on date and cause of death (in-hospital and out-of-hospital) during follow up the cohort was linked to the Dutch National Cause of Death Register. Cause of death in this registry is coded according to the 10th revision of the International Classification of Diseases and Related Health Problems (ICD-10) [15]. Follow-up data on mortality was collected until January 2011. Eighteen of the 405 patients (4%) could not be linked with the Death Register. For these patients information on cause and date of death was obtained by scrutinizing patient files of the general practitioners (maximum follow-up until June 2007) [16].

Information on non-fatal pulmonary endpoints (exacerbation of COPD or pneumonia) was also obtained by scrutinizing patient files of the general practitioners, including specialist letters and drug prescriptions. Data on exacerbations and pneumonia was gathered till the patient moved, died or the end of study (June 2007), whatever came first [16]. Exacerbation of COPD was defined as symptomatic deterioration requiring pulsed oral steroids or hospitalization for an exacerbation [11]. The diagnosis of ‘pneumonia’ was based on the general practitioner's diagnosis or hospitalizations for pneumonia.

### Covariates

As the association between resting heart rate and mortality or nonfatal pulmonary complications may be confounded by patient characteristics, we studied the influence of various covariates on the calculated associations. Potential confounders, measured at baseline, included age, sex, pack-years of smoking, COPD severity (the percentage of predicted forced expiratory volume in 1 second [FEV1] was used as a proxy), body mass index (BMI), co-morbidities (history of hypertension, diabetes mellitus, cardiovascular disease, hypercholesterolemia, or malignancies), and the use of medication (cardiovascular [β-blockers excluded], β-blockers, and respiratory drugs [β_2_-agonists, anticholinergics and inhaled corticosteroids]). Data on co-morbidities were acquired from patient files of the general practitioners. Smoking habits were obtained by a standardised questionnaire. BMI was calculated as weight (kg)/length^2^ (m^2^). Spirometric measurements were performed in all patients. A bronchodilator reversibility test was executed after inhalation of two puffs of 20 µg ipatropium bromide by inhalation chamber, after a time interval of at least 30 minutes.

Patients were grouped according to fulfilling the criteria of the Global Initiative for Chronic Obstructive Lung Disease (GOLD) for COPD (n = 244, 60%), or not. Patients fulfilled the GOLD criteria for COPD when the ratio of the post-dilatory forced expiratory volume in 1 second and the forced vital capacity ratio (FEV1/FVC) was <70%, either with or without complaints [1].

### Statistical analyses

Cox regression analysis was used to calculate the risk for mortality in relation to resting heart rate. We adjusted for two sets of confounders: 1) age and sex, 2) all covariates that influenced the association. All potential confounders that were univariately associated with the outcome and changed the β of the association with at least 10%, were included in the multivariable analyses. Heart rate was either categorised in steps of 10 beats per minute (bpm) and analysed as a continuous variable or dichotomized in the following categories: ≤80 bpm and >80 bpm. The group with a heart rate ≤80 bpm was taken as the reference category. A stratified analyses for mortality was performed regarding sex. The presence of interaction on a multiplicative scale between heart rate and sex was estimated by including the cross-product of the two factors as a variable in the model. A separate Cox regression analysis was performed to examine the association between heart rate and non-fatal pulmonary complications.

Sensitivity analyses were conducted to investigate whether the effect of misclassification of COPD and the use of β-blockers influenced the results, by repeating all analyses in subgroups with stricter inclusion criteria: first including only patients with COPD according to the GOLD criteria (n = 244), second including only patients with COPD according to the GOLD criteria who did not receive β-blockers (n = 219). All data were analysed using the statistical software package of SPSS (SPSS for Windows, version 14.0, SPSS Inc.).

## Results

The baseline characteristics of the participants are presented in table 1. The mean age of the patients at the start of the study was 73 (standard deviation [SD] 5) years, and 55% were male. Patients with a heart rate higher than 80 bpm were older and less often male, had a higher median pack-years of smoking, had a lower post-dilatory FEV1, and used more cardiovascular drugs, but less β-blockers than patients with a heart rate of 80 bpm or lower.

**Table 1:** Baseline characteristics of the 405 patients with a diagnosis of chronic obstructive pulmonary disease (COPD), divided in those with a heart rate of 80 bpm or lower versus those with a heart rate above 80 bpm.

Characteristics	All participants	Heart rate ≤80	Heart rate >80	P-value
	N = 405	N = 310	N = 95	
Male	221 (55%)	174 (56%)	47 (50%)	0.25
Age (years)	73 (5)	73 (5)	74 (6)	0.39
Smoking				
*Never smoked*	*104 (26%)*	*86 (28%)*	*18 (20%)*	
*Past or current smoker*				
*≤15 pack-years*	*83 (21%)*	*66 (21%)*	*17 (18%)*	
*>15 pack-years*	*198 (49%)*	*145 (47%)*	*53 (56%)*	
*Unknown number of pack-years*	*20 (5%)*	*13 (4%)*	*7 (7%)*	*0.15*
FEV1 (% predicted)	83 (26)	85 (26)	77 (24)	0.01
FEV1/FVC ratio	0.64 (0.14)	0.65 (0.14)	0.64 (0.15)	0.38
Body mass index (kg/m^2^)^1^	27 (4)	26 (4)	28 (4)	0.04
Mean systolic blood pressure (mmHg)	152 (18)	151 (18)	153 (20)	0.44
Mean diastolic blood pressure (mmHg)	84 (10)	83 (10)	86 (11)	0.002
COPD severity				
*No COPD according to GOLD criteria*	*161 (40%)*	*125 (40%)*	*36 (38%)*	
*Stage I*	*79 (20%)*	*66 (21%)*	*13 (14%)*	
*Stage II*	*120 (30%)*	*88 (28%)*	*32 (34%)*	
*Stage III–IV*	*45 (11%)*	*31 (10%)*	*14 (15%)*	*0.22*
History of				
*Diabetes mellitus*	*42 (10%)*	*28 (9%)*	*14 (15%)*	*0.11*
*Hypertension*	*145 (36%)*	*98 (32%)*	*47 (50%)*	*0.001*
*Cardiovascular disease* 2	*180 (44%)*	*137 (44%)*	*43 (45%)*	*0.85*
*- Ischaemic heart disease*	*126 (31%)*	*97 (31%)*	*29 (31%)*	*0.89*
*- Cardiac arrhythmias*	*40 (10%)*	*29 (9%)*	*11 (12%)*	*0.53*
*- Stroke*	*21 (5%)*	*13 (4%)*	*8 (8%)*	*0.10*
*Malignancies*	*32 (8%)*	*23 (7%)*	*9 (10%)*	*0.52*
*Hypercholesterolemia*	*45 (11%)*	*36 (12%)*	*9 (10%)*	*0.56*
Medication				
*Cardiovascular drugs* 3	*230 (57%)*	*158 (51%)*	*72 (76%)*	*<0.001*
*- 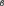 -blockers*	*47 (12%)*	*40 (13%)*	*7 (7%)*	*0.14*
*Inhalatory respiratory drugs*				
*- 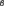 _2_-agonists*	*238 (59%)*	*176 (57%)*	*62 (65%)*	*0.14*
*- Anticholinergics*	*192 (47%)*	*138 (45%)*	*54 (57%)*	*0.04*
*- Corticosteroids*	*254 (63%)*	*189 (61%)*	*65 (68%)*	*0.19*
Heart rate (bpm)	71 (14)	65 (8)	91 (10)	<0.001
Mean RR interval length (ms)^1^	871 (165)	935 (131)	664 (63)	<0.001

Values are means (SD) for continuous variables, absolute numbers (percentages) for dichotomous variables and median (25–75 percentile) for skewed distributed variables.

SD: standard deviation, N: number, COPD: chronic obstructive pulmonary disease, FEV1: forced expiratory volume in 1 second, FVC: forced vital capacity, bpm: beats per minute, GOLD: global initiative for chronic obstructive lung disease, ms: milliseconds.

1 Body mass index: 4 missing, mean RR: 2 missing.

2 Including prior myocardial infarction, angina pectoris, coronary artery bypass grafting, percutaneous coronary intervention, atrial fibrillation, supraventricular tachycardia, ventricular fibrillation, ventricular tachycardia, other cardiac arrhythmias, stroke, transient cerebral ischemic attack, peripheral arterial disease, or aortic aneurysm.

3 Including diuretics, digoxin, calcium channel-antagonists, anti-arrhythmics, platelet aggregation inhibitors, ACE inhibitors, angiotensin II receptor blockers, nitrates and statins.

Participants were followed up on mortality for a median period of 7.0 years (range: 7 days to 9.0 years). During follow up 132 (33%) patients died (table 2). In 39 (30%) patients cardiovascular diseases were considered the cause of death, and in 36 (27%) respiratory diseases. In the subgroup of patients with COPD according to the GOLD criteria, 97 (40%) patients died. The overall mortality rate was 50/1000 patient-years (py, 95% CI 42–59). The cardiovascular mortality rate was 15/1000 py [95% CI 11–20]), and the respiratory mortality rate was 14/1000 py [95% CI 10–19]).

**Table 2:** Mortality and exacerbations/pneumonia in 405 patients with a diagnosis of chronic obstructive pulmonary disease (COPD), divided in those with a heart rate of 80 bpm or lower versus those with a heart rate above 80 bpm.

Characteristics	All participants	Heart rate ≤80	Heart rate >80	P-value
	N = 405	N = 310	N = 95	
*All-cause mortality*	*132 (33%)*	*87 (28%)*	*45 (47%)*	*<0.001*
Cardiovascular death	39 (10%)	24 (8%)	15 (16%)	
Respiratory deaths	36 (9%)	19 (6%)	17 (18%)	
Other causes	57 (14%)	44 (14%)	13 (14%)	<0.001
*Non-fatal pulmonary complication* 1	*179 (44%)*	*128 (42%)*	*51 (54%)*	*0.038*

Values are absolute numbers (percentages).

1 pneumonia and/or exacerbation.

The observed relative risk for all-cause mortality in the 405 COPD patients increased with 21% for every 10 bpm increase in resting heart rate (crude hazard ratio [HR]: 1.28 [1.14–1.43], p<0.001, adjusted HR: 1.21 [1.07–1.36], p = 0.002, table 3). Likewise, for every 10 bpm increase in resting heart rate, the observed risk of cardiovascular mortality increased with 43% (crude HR: 1.44 [1.18–1.75], p<0.001, adjusted HR: 1.43 [1.17–1.76], p = 0.001), and the observed risk of respiratory mortality increased with 51% (crude HR: 1.54 [1.26–1.89], p<0.001, adjusted HR: 1.51 [1.19–1.90], p = 0.001).

**Table 3:** Association of heart rate with all-cause, cardiovascular and respiratory mortality in 405 patients with a diagnosis of chronic obstructive pulmonary disease.

Heart rate	Person-years	Deaths	Mortality/1000 person-years (95%CI)	Crude HR (95%CI)	p value	Adjusted HR^1^ (95%CI)	p value	Adjusted HR (95%CI)	p value
*All-cause mortality*									
Continuous	2665	132	50 (42–59)	1.28 (1.14–1.43)	<0.001	1.27 (1.14–1.43)	<0.001	1.21 (1.07–1.36)^2^	0.002
≤80 bpm	2102	87	41 (33–51)	Reference		Reference		Reference	
>80 bpm	563	45	80 (59–106)	2.0 (1.4–2.9)	<0.001	1.9 (1.3–2.8)	<0.001	1.6 (1.1–2.3)^2^	0.013
*Cardiovascular mortality* 3									
Continuous	2665	39	15 (11–20)	1.44 (1.18–1.75)	<0.001	1.42 (1.16–1.75)	0.001	1.43 (1.17–1.76)^4^	0.001
≤80 bpm	2102	24	11 (7–17)	Reference		Reference		Reference	
>80 bpm	563	15	27 (15–43)	2.4 (1.3–4.6)	0.008	2.2 (1.1–4.2)	0.019	2.3 (1.2–4.5)^4^	0.017
*Respiratory mortality*									
Continuous	2665	36	14 (10–19)	1.54 (1.26–1.89)	<0.001	1.54 (1.25–1.89)	<0.001	1.51 (1.19–1.90)^5^	0.001
≤80 bpm	2102	19	9 (6–14)	Reference		Reference		Reference	
>80 bpm	563	17	30 (18–47)	3.5 (1.8–6.8)	<0.001	3.3 (1.7–6.4)	<0.001	2.8 (1.4–5.4)^5^	0.002

Heart rate was categorised in steps of 10 bpm, when analysed as a continuous variable. In total, 310 patients had a heart rate ≤80 bpm and 95 patients had a heart rate >80 bpm.

COPD: chronic obstructive pulmonary disease, HR: hazard ratio, CI: confidence interval, bpm: beats per minute.

1 Adjusted for sex, and age.

2 Adjusted for sex, age, pack-years of smoking, FEV1, and use of cardiovascular drugs (β-blockers excluded).

3 One patient was censored before the earliest event in this stratum occurred and therefore excluded from analysis.

4 Adjusted for sex, age, history of cardiovascular disease, use of cardiovascular medication (β-blockers excluded), and β-blockers.

5 Adjusted for sex, age, pack-years of smoking, and FEV1(% predicted).

A heart rate of more than 80 bpm was associated with a significant increased risk of death from all causes in COPD patients compared to a heart rate of 80 bpm or lower (adjusted HR: 1.6 [1.1–2.3], p = 0.013). The observed risk of cardiovascular and respiratory mortality were also increased in COPD patients with a heart rate higher than 80 bpm as compared to those with a heart rate of less than 80 bpm (adjusted HR: 2.3 [1.2–4.5], p = 0.017, 2.8 [1.4–5.4], p = 0.002, respectively).

Stratification according to sex showed a somewhat stronger effect of heart rate in women (crude HR: 1.37 [1.12–1.68], p = 0.002, adjusted HR: 1.28 [1.05–1.57], p = 0.016) than in men (crude HR: 1.23 [1.07–1.40], p = 0.004, adjusted HR: 1.15 [0.99–1.33],p = 0.076), but the interaction between sex and heart rate was not statistically significant on a multiplicative scale (p = 0.136).

Participants were followed for non-fatal pulmonary complications during a median period of 3.5 years (range: 2 days to 6.1 years). Forty-four percent of the patients experienced at least one episode of exacerbation of COPD or pneumonia during follow-up (n = 179). One patient did not have any follow-up data. The incidence of non-fatal pulmonary events was 145/1000 py (95%CI 120–168).

COPD patients with a heart rate of more than 80 bpm did not have an increased observed risk of pneumonia or exacerbation compared to patients with a resting heart rate of 80 bpm or lower (adjusted HR: 1.1 [0.8–2.0], p = 0.437, table 4). A non-significantly increased risk of a non-fatal pulmonary complication was observed in COPD patients with 7% for every 10 bpm of increase in heart rate (adjusted HR: 1.07 [0.96–1.18], p = 208).

**Table 4:** Association of heart rate with non-fatal respiratory complications (pneumonia or exacerbation) in 402^1^ patients with a diagnosis of chronic obstructive pulmonary disease.

Heart rate	Person-years	Pneumonia or exacerbation	Incidence/1000 person-years (95% CI)	Crude HR (95%CI)	P value	Adjusted HR^2^ (95%CI)	P value	Adjusted HR^3^ (95%CI)	P value
Continuous	1234	179	145 (120–168)	1.14 (1.03–1.26)	0.010	1.13 (1.03–1.25)	0.013	1.07 (0.96–1.18)	0.208
≤80 bpm	975	128	131 (110–156)	Reference		Reference		Reference	
>80 bpm	259	51	197 (148–257)	1.5 (1.1–2.0)	0.18	1.5 (1.1–2.0)	0.021	1.1 (0.8–1.6)	0.437

Heart rate was categorised in steps of 10 bpm, when analysed as a continuous variable. In total 310 patients had a heart rate ≤80 bpm and 95 patients had a heart rate >80 bpm.

COPD: chronic obstructive pulmonary disease, HR: hazard ratio, CI: confidence interval, bpm: beats per minute.

1 Two patients were excluded as they were censored before the earliest event occurred. In one patient we had no follow-up data.

2 Adjusted for sex, and age.

3 Adjusted for sex, age, pack-years of smoking, FEV1 (% predicted), use of cardiovascular drugs (β-blockers excluded), and β-blockers.

To account for the effect of misclassification of COPD and use of β-blockers, we performed sensitivity analyses by repeating analyses on all-cause mortality and exacerbations/pneumonia in 2 subgroups with different inclusion criteria, which showed similar results as the analyses of the tot group of 405 patients (Table S1). In the subgroup of patients with COPD according to the GOLD criteria (n = 244) the adjusted HR for all-cause mortality was 1.25 (1.07–1.45), p = 0.004, and for non-fatal pulmonary complications 1.07 (0.94–1.22), p = 0.202. In the subgroup of patients with GOLD-COPD who did not receive β-blockers (n = 219) the adjusted HR for all-cause mortality was 1.15 (0.99–1.35), p = 0.076, and for non-fatal pulmonary complications 1.08 (0.95–1.24), p = 0.249.

## Discussion

We showed that an increased resting heart rate is a strong and independent risk factor for all-cause mortality in elderly men and women with COPD, however, not on non-fatal pulmonary complications. Patients with COPD in general have a higher resting heart rate than patients without COPD [5], [7], and population-based studies clearly showed that elevated resting heart rate is associated with an increased risk of cardiac mortality [17]–[20]. A recent study of Jensen *et al.* confirmed that these results also apply to patients with COPD. In a prospective study of almost 17,000 subjects and 2645 COPD patients they showed that patients with COPD and a heart rate ≥85 bpm had an increased risk of all-cause and cardiovascular mortality, compared to those with a heart rate of less than 64 bpm (adjusted HR 1.51 [1.43–1.60], 1.57 [1.45–1.71], respectively) [21].

To the best of our knowledge, we are the first who showed that an increased resting heart rate did not result in non-fatal pulmonary complications in patients with COPD. This may indicate that the increased mortality observed in COPD patients with high resting heart rates is mainly driven by non-pulmonary causes. Especially cardiovascular diseases could account for this ‘mismatch’ because patients with COPD frequently have concurrent cardiovascular disease, often undetected or latent [10], [11]. Moreover, both COPD and cardiovascular diseases share a relation with cigarette smoking, a well-known cause of endothelial dysfunction and risk factor for cardiovascular events.

The relation between resting heart rate and respiratory mortality is probably overestimated. Previous studies showed overestimation of COPD as the cause of death mentioned on death certificates [22]. An effect that certainly is even stronger when patients during life are known with a pulmonary disease.

A potential mechanism of the increased heart rate in patients with COPD is autonomic dysfunction. Automatic dysfunction may be triggered by longstanding periodically hypoxemia. Autonomic dysfunction contributes to the development of cardiovascular diseases, especially arrhythmias, abnormal conduction and ectopic beats [9], but possibly also heart failure [11]. In addition, the use of β_2_-agonists could contribute to the elevated resting heart rate, especially the short-acting β_2_-agonists. As inhaled β_2_-agonists are central to symptom management in COPD, their use could be an additional explanation of the increased heart rate found in COPD patients as compared to an age-matched population at large.

Traditionally, β-blockers, having an opposite effect to β_2_-agonists, have been considered contra-indicated in patients with COPD. The first Cochrane review of Salpeter *et al.* was a cornerstone study because it showed that cardio-selective β-blockers were well tolerated by patients with COPD, without adverse effects on FEV1, respiratory symptoms or response to β_2_-agonists [23]. Recent studies showed that beyond safety, long-term treatment with (cardioselective) β-blocking agents even may improve survival of patients with COPD [11], [24], and a reduction in exacerbations [11]. Although, counterintuitive on first glance, combining β_2_-agonists and β-blockers seems a good treatment option when both drugs are indicated, because of counterbalancing possible negative effects of each drug [25]. β-blocking agents are known to diminish sympathetic nerve system activity, and thus reduce heart rate and this mechanism may reduce mortality in patients with cardiovascular disease and in those with COPD [11], [26], [27]. Randomized controlled trials are needed to confirm if β-blockers, or other heart rate reducing drugs, are beneficial in reducing fatal and nonfatal complications in patients with COPD. Importantly, however, β-blockers could be beneficial on exacerbation because of other mechanism than reduction of heart rate, because we showed that heart rate had no significant effect on non-fatal pulmonary complications. In addition, exercise therapy might achieve a lower resting heart rate [28].

Some potential limitations of the present study should be taken into account. Only 60% of the 405 patients with a general practitioners diagnosis of COPD had COPD according to the GOLD criteria (post-dilatory FEV1/FVC<70%). Importantly, however, a sensitivity analysis in those 244 patients showed similar results as presented in the whole group on both endpoints. Another limitation could be overestimation of pulmonary cause of death on the death certificates because the cause of death of the National Cause of Death Register was not validated by medical records or autopsy reports, although, several studies have shown that in general the validity of the cause of death registration of the Dutch National Cause of Death Register is adequate [29]. Finally, we defined exacerbation as recommended (symptomatic deterioration requiring steroids use or hospitalization), with the exception that we did not included ‘antibiotic use only’ [30]. In the Netherlands it is common practice and advocated by Dutch guidelines to treat exacerbations with short-course corticosteroids, and consider to add antibiotics only when a bacterial infection is suspected.

The strength of our study is that we could extensively adjust for potential confounding factors, as we had much information on important potential confounding factors, including detailed information on pack-years of smoking and pulmonary function test parameters. Another advantage of this general practitioners cohort is that it can be considered as population-based, as all community-dwelling persons with COPD, including those treated by a pulmonologist, were included.

## Conclusions

Increased heart rate is a strong and independent risk factor for all-cause mortality in patients with a diagnosis of COPD. There was, however, no significant association between heart rate and major respiratory complications, and this may indicate that the increased risk of mortality of patients with COPD is determined by non-pulmonary causes. Especially cardiovascular diseases could account for these findings because of the high concurrency with COPD and its mutual relation with smoking. Future randomized controlled trials are needed to investigate whether heart-rate lowering agents, notably β-blockers, would be worthwhile for patients with COPD.

## Supporting Information

Table S1 Sensitivity analyses. The association of heart rate and mortality or non-fatal pulmonary complications in 2 subgroups: 1) COPD patients with COPD according to the GOLD criteria, 2 COPD patients with COPD according to the GOLD criteria who did not use betablockers.
(DOCX)
