# Supplementary material for: Resting Heart Rate Is a Risk Factor for Mortality in Chronic Obstructive Pulmonary Disease, but Not for Exacerbations or Pneumonia
Source: PLoS One. 2014 Aug 26;9(8):e105152. doi: 10.1371/journal.pone.0105152 (PMC4144884; doi:10.1371/journal.pone.0105152)
Supplement: Table S1 — Sensitivity analyses. The association of heart rate and mortality or non-fatal pulmonary complications in 2 subgroups: 1) COPD patients with COPD according to the GOLD criteria, 2 COPD patients with COPD according to the GOLD criteria who did not use betablockers. (DOCX) [file pone.0105152.s001.docx]

**Supplementary table S1.** Sensitivity analyses. The association of heart rate and mortality or non-fatal pulmonary complications in 2 subgroups:

1) COPD patients with COPD according to the GOLD criteria, 2 COPD patients with COPD according to the GOLD criteria who did not use betablockers.

| **Heart rate** | **Person-years** | | **Outcome** | | **Incidence/1000 person-years (95%CI)** | **Crude HR**  **(95%CI)** | **p value** | **Adjusted HR^1^ (95%CI)** | **p value** | **Adjusted HR (95%CI)** | **p value** |
| --- | --- | --- | --- | --- | --- | --- | --- | --- | --- | --- | --- |
| **COPD patients with COPD according to the GOLD criteria ( n=244)** | | | | | | | | | | | |
| *All-cause mortality* | | |  | |  |  |  |  |  |  |  |
| Continuous | 1562 | | 97 | | 62 (51-75) | 1.29 (1.11-1.49) | 0.001 | 1.29 (1.11-1.50) | 0.001 | 1.25 (1.07-1.45)^2^ | 0.004 |
| ≤80 bpm  >80 bpm | 1223  339 | | 65  32 | | 53 (41-67)  94 (66-132) | Reference  1.8 (1.2-2.8) | 0.005 | Reference  1.8 (1.2-2.7) | 0.009 | Reference  1.6 (1.0-2.4)^2^ | 0.038 |
| *Non-fatal respiratory complications* | | | | | |  |  |  |  |  |  |
| Continuous | 657 | 128 | | 195 (163-231) | | 1.16 (1.02-1.32) | 0.027 | 1.16 (1.02-1.32) | 0.028 | 1.07 (0.94-1.22)^3^ | 0.293 |
| ≤80 bpm  >80 bpm | 519  138 | 92  36 | | 177 (144-216)  261 (186-357) | | Reference  1.4 (1.0-2.1) | 0.061 | Reference  1.5 (1.0-2.1) | 0.060 | Reference  1.2 (0.8-1.7)^3^ | 0.450 |
| **COPD patients with COPD according to the GOLD criteria, who did not use a beta-blocker ( n=219)** | | | | | | | | |  |  |  |
| *All-cause mortality* |  |  | |  | |  |  |  |  |  |  |
| Continuous | 1176 | 91 | | 77 (63-95) | | 1.23 (1.06-1.44) | 0.008 | 1.23 (1.06-1.44) | 0.008 | 1.15 (0.99-1.35)^2^ | 0.076 |
| ≤80 bpm  >80 bpm | 889  287 | 61  30 | | 69 (53-88)  105 (72-147) | | Reference  1.6 (1.1-2.5) | 0.028 | Reference  1.6 (1.0-2.4) | 0.047 | Reference  1.3 (0.8-2.1)^2^ | 0.219 |
| *Non-fatal respiratory complications* | | | | | |  |  |  |  |  |  |
| Continuous | 570 | 121 | | 212 (177-253) | | 1.14 (1.00-1.30) | 0.053 | 1.14 (1.00-1.31) | 0.052 | 1.08 (0.95-1.24)^4^ | 0.249 |
| ≤80 bpm  >80 bpm | 437  133 | 85  36 | | 195 (156-239)  271 (192-371) | | Reference  1.4 (0.9-2.0) | 0.116 | Reference  1.4 (0.9-2.0) | 0.112 | Reference  1.2 (0.8-1.8)^4^ | 0.386 |

COPD: chronic obstructive pulmonary disease, GOLD: global initiative for chronic obstructive lung disease, HR: hazard ratio, CI: confidence interval, bpm: beats per minute, N: number.

^1^ Adjusted for sex and age.

^2^ Adjusted for sex, age, FEV1, pack-years of smoking, and use of cardiovascular drugs (no beta-blockers)

^3^ Adjusted for sex, age, pack-years of smoking, FEV1 (% predicted), use of cardiovascular drugs (β-blockers excluded), and β-blockers

^4^ Adjusted for sex, age, pack-years of smoking, FEV1 (% predicted), and use of cardiovascular drugs (β-blockers excluded)
